# Cooperative UAV–UGV Autonomous Power Pylon Inspection: An Investigation of Cooperative Outdoor Vehicle Positioning Architecture

**DOI:** 10.3390/s20216384

**Published:** 2020-11-09

**Authors:** Alvaro Cantieri, Matheus Ferraz, Guido Szekir, Marco Antônio Teixeira, José Lima, André Schneider Oliveira, Marco Aurélio Wehrmeister

**Affiliations:** 1 Applied Robotics and Communication Laboratory—ARCLab—Information and Communication Group, Federal Institute of Paraná, Curitiba, Paraná 3100, Brazil; 2 Graduate Program in Electrical And Computer Engineering (CPGEI), Universidade Tecnológica Federal do Paraná, Curitiba, Paraná 3131, Brazil; matf.ferraz@gmail.com (M.F); guidoszekir@gmail.com (G.S.); marcoteixeira@alunos.utfpr.edu.br (M.A.T.); andreoliveira@utfpr.edu.br (A.S.O.); wehrmeister@utfpr.edu.br (M.A.W.); 3 CeDRI—Research Centre in Digitalization and Intelligent Robotics, Instituto Politécnico de Bragança and INESC TEC, R. Dr. Roberto Frias 400, 4200 Porto, Portugal; jllima@ipb.pt

**Keywords:** cooperative UAV–UGV energy pylon inspection, cooperative vehicle position sensing, autonomous power line inspection

## Abstract

Realizing autonomous inspection, such as that of power distribution lines, through unmanned aerial vehicle (UAV) systems is a key research domain in robotics. In particular, the use of autonomous and semi-autonomous vehicles to execute the tasks of an inspection process can enhance the efficacy and safety of the operation; however, many technical problems, such as those pertaining to the precise positioning and path following of the vehicles, robust obstacle detection, and intelligent control, must be addressed. In this study, an innovative architecture involving an unmanned aircraft vehicle (UAV) and an unmanned ground vehicle (UGV) was examined for detailed inspections of power lines. In the proposed strategy, each vehicle provides its position information to the other, which ensures a safe inspection process. The results of real-world experiments indicate a satisfactory performance, thereby demonstrating the feasibility of the proposed approach.

## 1. Introduction

Power line inspection is an important task performed by energy distribution companies. This inspection is essential to ensure steady energy delivery and avoid service interruptions. The inspection process involves different tasks, depending on the objective of the operation. The Brazilian National Electrical Energy Agency broadly classifies the activities of transmission line inspection as corresponding to terrestrial, aerial, or detailed inspection [1]. Terrestrial inspection commonly occurs over an extended range of the transmission line through foot patrol. The objective is to evaluate the general condition of the transmission line, pylon base stability, occurrence of soil erosion, access to the structures, and proximity of vegetation to the cables, among other factors. Aerial inspection involves a long-range visual evaluation of the transmission lines, thereby providing an overview of the metallic structure conditions and allowing the identification of the damages and defects in the components. The objective of a detailed inspection is to identify small defects in the components, which cannot be easily detected in extended range inspection, through a close up visual operation. In general, this inspection is carried out by a technician climbing onto the energy pylon, with line-following robots or a small multi-rotor aircraft. This process is technically demanding and presents a substantial risk for the human operator and equipment, as it is usually executed with the line energized.

In recent decades, detailed inspection has been increasingly performed using small aircraft, specifically, unmanned aerial vehicles (UAVs), instead of by humans. UAVs can carry an extensive range of small sensors, such as regular, thermal, and multi-spectral cameras, 3D light detection and ranging (LIDAR) equipment, and radio frequency (RF) discharge detectors, which can enhance the data quality and process efficacy [2]. In such scenarios, a pilot remotely controls the flight of the aircraft around the energy pylon and power lines, following regular UAV inspection operation protocols, and the energy technician observes the images to identify any likely damages or defects in the structure. The position control of the UAV must be robust and accurate to avoid collision with the structure; thus, the pilot must maintain a visual link with the aircraft during the process. Furthermore, the energy power structure inspection presents several specific challenges:

- Owing to the extensive range of inspection segments, a long-duration energy supply must be provided to the small aircraft.
- Owing to the high complexity of the pylon metallic structures and energy cables, conventional sensors may find it challenging to detect obstacles.
- The electrical and magnetic fields present around the structure owing to the high voltage energy transmitted can likely interfere with the UAV’s navigation hardware and sensors [3].
- Foreign elements may be present in the vicinity of the distribution line, such as trees and buildings, which may lead to accidents.
- Obtaining physical access to the pylon area may be difficult owing to restricted areas or dangerous terrain; thus, the aircraft must take-off from a safe distance.
- It is challenging to fly near the pylon (2–5 m) to obtain images and data for analysis.
- Maintaining the position and orientation of the aircraft during the image acquisition process may be challenging owing to the presence of gusts and uncertainty in the standard positioning sensor data.

To alleviate the risks in such operations, a promising approach is to use an autonomous UAV flight system. Nevertheless, precise positioning must be realized to implement autonomous operation. A typical approach to provide accurate positioning data to a UAV is through the Differential Global Navigation Satellite System (DGNSS). In this approach, a static GNSS base reference (base station) sends the position data to the mobile module (rover) and calculates the position with an accuracy down to the centimeter level. This technique is a highly effective solution for providing precise horizontal positioning for small aircraft; however, it is effective only under specific operational conditions. The presence of obstacles, base antenna shadowing by constructions or trees, interference communication links among the modules [4], and cloud conditions may degrade system accuracy [5]. Thus, it is necessary to develop complementary positioning solutions to integrate into the UAV control system.

In this regard, in this work a cooperative UAV–unmanned ground vehicle (UGV) positioning architecture was investigated, especially for carrying out detailed inspections of power distribution infrastructure. An augmented reality tag (AR-Tag) was used to create a relative positioning reference, allowing the exchange of position data between the vehicles. Real-world experiments were performed to evaluate the feasibility of the proposal by determining the accuracy of the acquired position data during the flight and the influences of the environmental conditions, camera performance, and AR-Tag operation conditions on the accuracy.

## 2. Related Works

Autonomous UAV-based inspection has gained considerable attention in the last decade due to the developments in platforms, process and control hardware, and sensor technology for small aircraft. The literature presents an extensive number of works in this specific area. In the paper [6], the authors reviewed the potential of these aircraft for civil applications, indicating especially the power line distribution sector as one of high interest. However, one of the main challenges for the autonomous UAV operation is to provide accurate position data to the control algorithm. A possible approach to developing UAV positioning systems is based in computer vision algorithms that use visual clues present in the environment to calculate the position and orientation of the aircraft. In the paper [7] a review of computer vision algorithms for UAV applications, including descriptions of works related to power line inspections, is presented. In the conclusion section, this paper cites some challenges to applying these algorithms in outdoor tasks, especially the high sensitivity to lighting conditions. In the work [8], the authors review vision control for UAVs focused on infrastructure inspection applications. The paper analyses works that apply optical flow algorithms, visual surveying algorithms, feature detection methods, and simultaneous localization and mapping (SLAM), and affirms that "each method is suitable for specific types of environments and target objects." This affirmation indicates that UAV vision control still requires significant research, depending on the specific inspection application. In the paper [9] the authors present an extensive review of automatic vision-based power line inspection techniques. The authors conclude that "no high-speed, fully autonomous, vision-based navigation for power line inspection has been successfully developed," indicating that further research for solving practical problems related to such applications is still necessary. The specific problem of calculating the UAV’s position relative to the power pylon has been investigated in some works. In the paper [10], an algorithm to measure the distance between a UAV and an electric pylon was developed based on monocular camera images. In the study [11], a point-line-based SLAM technique was used to calculate the center of the pylon structure based on image processing. To implement visual odometry algorithms, suitable visual reference points must be provided in the terrain, which incurs high computational costs. These aspects have been discussed in detail in a previous work [12]. The processing demands of visual positioning algorithms can be alleviated by applying artificial visual marks, known as tags. In most cases, a tag refers to a drawing that presents specific visual characteristics, thereby improving the quality of the visual clues for image processing. Examples of this are [13,14,15,16].

### 2.1. Cooperative UAV–UGV Architectures

Use of cooperative UAV–UGV autonomous systems is a powerful approach for several applications, including inspection tasks. The specific characteristics of each vehicle provide a complementary gain in capability and enhance data collection, payload capability, and the maneuverability and range of operation of the vehicles. Many review studies on cooperative UAV–UGV applications have been presented so far, which describe the main application domains of such cooperative systems, such as SLAM, positioning, inspection, transportation, formation control, and swarming [17,18,19,20].

Some researchers proposed a visual feedback cooperative system between an AR-Drone quadcopter and a Pioneer UGV [21]. The system involved an automatic obstacle avoidance algorithm, in which the UAV captured images of a pre-assigned marker fixed on the top of the obstacle to reduce the image processing complexity. Another mark was fixed at the top of the UGV to provide a visual clue for the UGV position calculation algorithm. When the UGV reached an obstacle, the collision avoidance algorithm executed the route deviation necessary to lead the UGV to a path surrounding the obstacle area. Furthermore, an ArUco AR-Tag [22] placed at the top of an autonomous ground vehicle was used to provide visual position feedback to a UAV [23]. The UAV took off from the UGV body and autonomously followed the ground vehicle. An operator set the path of the UGV, and the autonomous algorithm maintained the UAV above it. At the end of the displacement, the UAV landed at the top of the UGV. A cooperative path planning algorithm, based on images collected by a UAV and a probabilistic roadmap path calculation, has also been proposed in [24]. Nevertheless, information regarding the image processing and the real-world data collection and experiments was not presented in the paper. In the work [25], a cooperative collision avoidance arrangement was developed, in which a UAV first performed an aerial mapping of the terrain to provide the obstacle positions to a path following UGV. The UGV was equipped with an RTK-GPS to provide high-accuracy positioning data to the control system. When the UGV reached an obstacle, an avoidance algorithm was executed. Some researchers proposed a cooperative obstacle detection and path planning arrangement using a UAV equipped with a down-point camera to capture images of the terrain and obstacles [26]. An image processing algorithm calculated the obstacle positions and performed A* path planning for the UGV. In addition, a cooperative target-following architecture was developed in [27]. A UGV followed an object covered with AprilTag markers, and the UAV followed the UGV through another AprilTag marker placed on the roof of the UGV. Researchers have developed a cooperative positioning system between a UAV and three small UGVs by using AR-Tags to create a relative reference; see [28]. This paper presented a preliminary investigation of our working group, in which this architecture was used to provide a cooperative reference between the air and ground autonomous vehicle applications. The architecture was evaluated in a simulation environment, and satisfactory results were obtained with the UAV providing the position data to control the UGV’s path.

### 2.2. Overall Analysis and Contributions of the Present Work

A cooperative UAV–UGV inspection architecture is presented in this study. Based on the above literature review, it can be concluded that there are gaps in the state-of-art with regard to UAV-based detailed inspection problems of power pylons. To the best of our knowledge, none of the studies that used a cooperative UAV–UGV architecture have applied it to power pylon or power line inspection. Moreover, the cooperative positioning method can also be considered to be novel. To present a comparative review, Table 1 presents an overview of the related works and compares them to our proposal.

This work’s key contribution is the investigation of a new collaborative positioning system architecture to realize power line pylon inspection by providing redundant data to increase the safety of the autonomous displacements of the UAV and UGV during the inspection process. We also investigated a complementary positioning method for power line inspection applications to be used when the DGNSS method fails. Visual positioning methods are susceptible to visual obstacles in every situation. Besides, the RTK-GPS is the primary positioning system used by the UAV in the proposal. In this study, the UGV uses RTK-GPS data when the UAV is carried by it to the nearby pylon. UGV visibility presents some limitations because of obstacles in the field. Thus, the method proposes that the UGV seeks the UAV so that it is always visible to the aircraft, thereby preventing the aforementioned problems. The advantages of the proposed method are as follows:

- Simple application, based on low-cost commonly available sensors resources (RGB camera, IMU).
- Compared with IMU and RTK-GPS sensors, the position sensing method is robust against electric and magnetic field disturbances in the inspection area [4,30].
- The cooperative inspection platform can enhance the capability of inspection data acquisition, allowing the use of multiple sensors embedded in each vehicle in a cooperative arrangement, such as optical zoom sensors, thermal and multi-spectral image sensors, high range LIDAR, and RF spark detectors, thereby generating a collaborative sensing inspection data set.

The limitations and assumptions of the method are as follows:

- A viable terrain to realize the UGV path following may not be available in all energy power sites.
- Due to the low accuracy of AR-Tag position readings at long distances, a large tag must be used to provide accurate data.

It is important to reinforce that this paper presents the first part of an extensive research project, including power line following and multiple pylon inspection processes. This long-range collaborative inspection demands additional intelligent algorithms to face the common problems encountered in practice, including collision avoidance and path planning. The development of these solutions is a work-in-progress for our research group. In this paper, the group chose to work in a particular set of controlled conditions as a preliminary approach to evaluate the main proposal of the collaborative positioning system.

As the energy power inspection process is similar to that of other structures, the proposed architecture can be extended to similar applications, such as the inspections of buildings, bridges, silos, cell towers, and dams. To the best of our knowledge, the proposed approach is the first of its kind among the available cooperative inspection systems.

## 3. Proposed Architecture of UAV–UGV Cooperative Inspection

**Figure 1:**
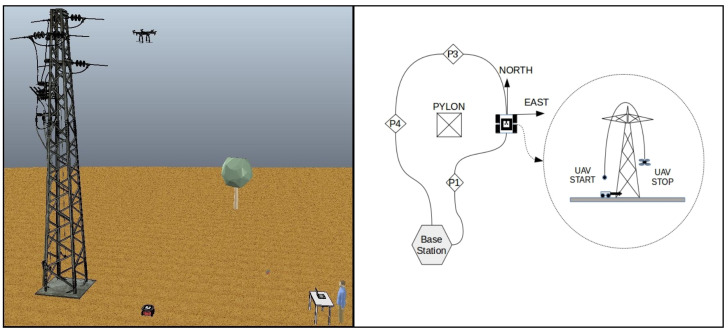
Overview of the architecture.

The system architecture is composed of a small quadrotor aircraft, a small UGV, a base station computer, and a RTK-GPS system. The agents collect data and send them to the base station, where the necessary calculations are performed. The base station defines the behavior of each vehicle and sends the position control data to the vehicles. Figure 1 shows an overview of the architecture.

All the architecture components use the Robot Operating System (ROS) to exchange information. ROS is an open-source, flexible, robust set of tools that provides information exchange standard messages between robots, sensors, processing hardware, software, and other robot tools [31].

The validation experiments were implemented using a Parrot Bebop drone with an embedded Emild Reach RTK-GPS module. The Bebop drone is suitable for autonomous algorithm development because it offers a stabilized flight performance and an embedded Full-HD resolution camera. The camera gimbal stabilization minimizes the image shifts during the data acquisition. The Emild Reach RTK modules [32] represent a low-cost small module RTK solution that allows the realization of autonomous UAV flight. The modules offer a Wi-Fi embedded link to communicate with the base station computer. The range of this link depends on the Wi-Fi base antenna gain. A regular notebook hardware offers a 500.0 m radius range without signal amplification.

The UGV is a Pioneer P3 four-wheel vehicle, controlled by an Intel Core i5 processor, with an 8 GB RAM running Ubuntu 16.4 LTS and ROS Kinect, developed by Generation Robots [33]. The vehicle is ROS compatible and embedded with sensors such as sound navigation and ranging (SONAR) and a Hokuyo URG-04-LX LIDAR sensor fixed in the front of the vehicle frame, allowing obstacle detection.

A computer running Ubuntu 16.4 LTS and ROS Kinect was used as the base station. The computer system includes an Intel Core i7 processor with 16 GB RAM and an Intel^®^ (Santa Clara, CA, USA) HD Graphics 520 (Skylake GT2) board. The base station runs the ROS Bebop Autonomy package [34] to realize the communication and control of the drone by using ROS nodes.

The communication between the agents and base station was implemented through three Wi-Fi network links, at frequencies of 5.8, 2.4, and 2.4 GHz from Bebop, RTK-GPS module, and Pioneer P3, respectively. The base station concentrates all the ROS messages and sends commands to the agents to execute their activities.

**Figure 2:**
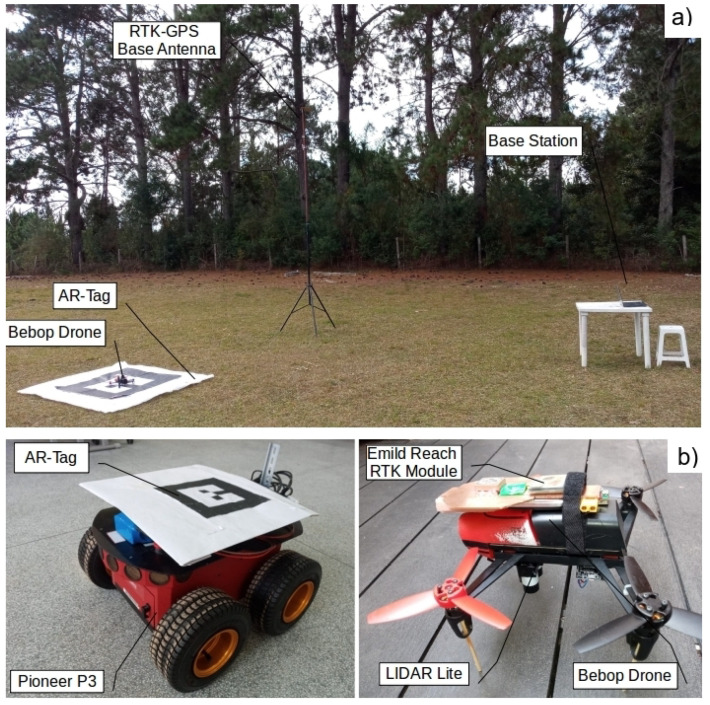
Architecture components. (**a**) Inspection site: base station, RTK-GPS, and UAV. (**b**) Detail of the Bebop drone with RTK-GPS module and Pioneer P3 with the augmented reality tag (AR-Tag).

The Ar-Track Alvar Augmented Reality solution [35] was used to implement the visual position calculations. The tool provides flexible usage and excellent computational performance. The Ar-Track Alvar package, developed by Scott Niekum, is ROS compatible and can minimize the implementation time. The package captures the AR-Tag images and publishes the position and orientation data on the *ar_pose_marker* ROS Node. Figure 2a details the architecture components, except the UGV. Details of the Bebop drone with the RTK-GPS module and LIDAR-Lite sensor are shown in Figure 2b, including the Pioneer UGV.

### 3.1. Inspection Procedure Parameters

The inspection procedure that serves as a reference for this research is based on technical information exchange with the local energy distribution company. Using this framework, the operational parameters of the UAV inspection system can be clearly defined, namely, the safe distance between the tower and UAV, average flight height, and navigation velocity during the inspection. Table 2 lists the technical parameters considered in this work.

**Figure 3:**
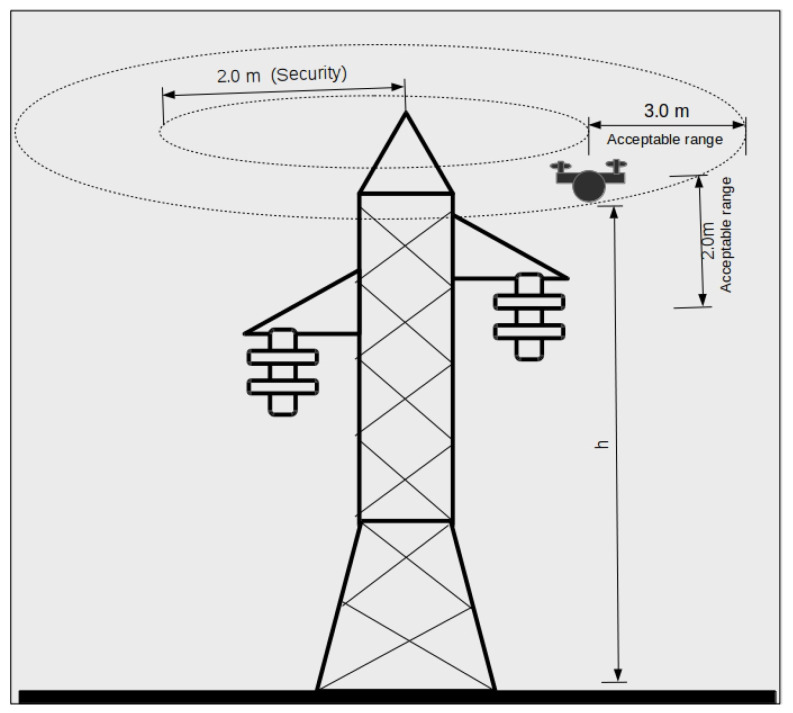
Autonomous flight limits for safe operation.

These parameters were defined based on the practical experience of engineers involved in UAV energy power inspection. In general, the inspection process consists of capturing the images of the structure and components in various sites and line segments using commercial UAVs. To a significant extent, the pilot’s expertise determines the level of safety of the inspection tasks; however, in some scenarios, the environmental conditions may pose a significant safety threat. It is a common perception among professionals that an autonomous flight solution could notably enhance the process safety. The autonomous flight limits for safe operation are specified in Figure 3.

It must be noted that these limits take into account the expertise of the operation engineer to recreate the same conditions for the autonomous flight as those present in a human-controlled flight in such inspection processes.

### 3.2. Inspection Process

This subsection describes the inspection process used in this study. Although the focus of this work is the evaluation of the positioning system, the description of the complete inspection process is important to understand the overall functioning of the system. To execute an inspection procedure, the UAV and UGV start at the base station position. The base station is where the supervising equipment, RTK-GPS base antenna, and central control of the system are located. For the first displacement from the base station to the nearby pylon, the UAV is carried by the UGV to save battery. To execute this displacement, the UGV uses the RTK-GPS data received by the UAV to feed the path following control algorithm, enhancing the vehicle position information’s accuracy.

When the UGV reaches the first inspection point around the pylon, the UAV takes off and performs an inspection path in the face of the pylon. This path is programmed carefully to ensure that the down-pointing camera of the UAV maintains a visual link with the AR-Tag placed at the top of the UGV. If the RTK-GPS module exhibits a loss of accuracy for any reason, the AR-Tag offers a redundant position reference to the UAV displacements, thereby enhancing the safety of the inspection process. The UAV captures the pylon images from the top, and the UGV simultaneously captures the image and data information from the base of the pylon. After the UAV finishes the inspection of a pylon segment, re-positioning of the UGV begins. At this time, positioning data are provided to the UGV by the AR-Tag reference system. The UAV remains in static flight above the desired end-point and sends a message to the ground vehicle to initiate its movement. The UGV receives the relative position data obtained from the AR-Tag tool and follows the UAV to immediately reach the point below it. If the vehicle encounters an obstacle, it performs a detour process based on readings from its frontal LIDAR. After reaching the final position with a preset position tolerance, the UGV stops moving, and the UAV begins a new pylon segment inspection. This process repeats until the UAV and UGV cover all the inspection points programmed in the path. After the last inspection point, the UAV reaches the UGV position and lands on its roof. The UGV returns to the base station carrying the UAV.

RTK-GPS provides the primary position reference of the UAV during the pylon inspection process. In this case, the AR-Tag is a redundant position system for the UAV displacements. Otherwise, the UGV position system considers the UAV/AR-Tag position information for all the pylon inspection displacements, and this framework represents the primary positioning system for the ground vehicle. The architecture generates a swinging positioning reference, enabling cooperative position data acquisition from each vehicle and the constant exchange of this information among them. Sharing the RTK-GPS sensor when the vehicles are coupled is key to decreasing the hardware necessary to implement the solution.

### 3.3. Referential Systems

As different referential systems work to control each vehicle, it is necessary to set the origin point to execute the reference transformations. The RTK base antenna is set as the origin (0,0,0) of the system, and the UAV position is calculated based on this point. It is vital to precisely set the antenna’s position relative to the power pylon before commencing the inspection process. This setup is performed using the geo-referenced positions of the pylons present on the energy company’s database.

The UGV referential system is set in accordance with the UAV position during the inspection process, and therefore, it represents a moving referential system. In each displacement, the UGV searches the position directly below the UAV, considering it as the (0,0) point. The UGV path is always set by the position of the UAV, in a "following the lead" process. This framework ensures that the ground vehicle remains below the UAV during the entire inspection process, maintaining tag visibility to serve as an optional reference point to the aircraft when a new inspection stage begins.

**Figure 4:**
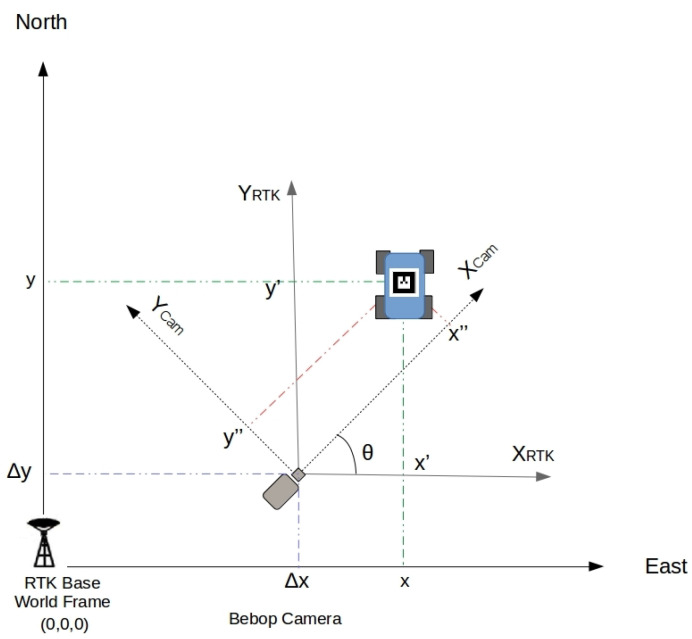
Reference corrections in the x–y plane.

Considering that the AR-Tag reference system is associated with the UAV camera axis, the relative orientation between the UAV camera and AR-Tag placed on top of the UGV when the vehicles are not aligned with one another creates an error in the tag position reading related to the world coordinate axis. Figure 4 represents the world axis with the origin at the RTK base, the UGV axis, and the Bebop drone camera axis.

The UGV position is read by the AR-Tag visual algorithm using the Bebop camera images. It is necessary to correct the offset (Δx, Δy) and the camera rotation *θ* to match the camera axis with the world axis. The camera angle correction is calculated with a 2D standard rotation matrix algorithm using the AR-Tag horizontal orientation information. Equation (1) presents the rotation and offset transformations.

(1) $$\left[\begin{array}{c} x\\ y \end{array}\right]=\left[\begin{array}{c} x^{\prime \prime }\\ y^{\prime \prime } \end{array}\right]*\left[\begin{array}{c} \cos\theta -\sin\theta \\ \sin\theta +\cos\theta \end{array}\right]+\left[\begin{array}{c} \Delta x\\ \Delta y \end{array}\right]$$

## 4. Experimental Setup

A set of real-world experiments was conducted to investigate the accuracy of the AR-Tag position and orientation readings in the proposed scheme. The objective was to estimate the error of the AR-Tag position and orientation readings in an outdoor environment under flight conditions and to evaluate the UGV control algorithm under the actual application conditions. A link for a Youtube video presenting the experiments is available in the Supplementary Materials section.

The Emlid Reach^TM^ RTK-GPS worked as a ground truth positioning system for the outdoor measurements. A LIDAR Lite sensor pointed to the ground provided the ground truth to the height measurements. The measurement site was an open field with no natural and artificial obstacles to the satellite visibility, which facilitated the realization of a proper reception signal to the base station and rover receiver installed in the UAV.

The data exchange between the RTK modules and base station computer was realized using the Reach RTK ROS Node Package [36]. The Bebop Autonomy ROS package [34] created the data exchange channel between Bebop and the base station. The package created nodes to publish the commands and subscribed to the data information, such as the camera captures and odometry data. The package limited the camera video stream to 640×368−30 Hz.

The Pioneer P3 vehicle involved an embedded PC that executed a regular PID position control algorithm. A *geometry_msgs:Twist* ROS message sent by the base station provided the position and orientation data for the UGV guidance. This data were extracted from the AR-Tag position readings.

A base PC worked as the ROS master, receiving the node data from all the agents and running the control and calculation codes. All the ROS nodes were captured simultaneously to synchronize the data collected and recorded in the ROS bag files. Equations (2) and (3) were used to calculate the absolute error mean and standard deviation for each group of samples, respectively.

(2) $$\bar{Error}=\sum_{i=1}^{n}\frac{p-p^{\prime }}{n}$$

(3) $$\sigma =\sqrt{\sum_{i=1}^{n}\frac{p-p^{\prime }}{n-1}}$$

where *p*’—read value, *p*—real value, and *n*—number of samples.

### 4.1. AR-Tag Position and Orientation Error Estimation in an Outdoor Environment

The AR-Tag Alvar tool is primarily applied in augmented reality systems; however, its flexibility makes it suitable for use in robot position applications. Nevertheless, the use of these tags in real-world outdoor environments presents certain challenges in the form of long-distance readings, lighting conditions, and shadowing of the tag.

An experiment was performed to evaluate the use of the tag in outdoor long-distance conditions. The Bebop drone was placed in a fixed position over a table, and the tag was fixed on a tripod. A 30.0-m-long measurement tape with an accuracy of 0.5 cm was fixed on the ground to serve as a distance reference. The error position and orientation were measured three times for each meter. The process was repeated using tags of two different sizes (50.0 and 90.0 cm). Camera resolutions of 640×368 pixels (Bebop autonomy resolution), 1280×720 pixels, and 1920×1080 pixels were considered. For the latter two resolutions, the tag images were recorded through Bebop and post-processed. The following illumination conditions were considered: (a) direct sunlight, (b) indirect sunlight, (c) cloudy, and (d) direct sunlight with partial tag shadowing. Table 3 presents the experiment data.

Four hundred samples were captured for each measurement. Equations (2) and (3) were used to calculate the absolute error, mean, and standard deviation for each group of samples, respectively.

**Figure 5:**
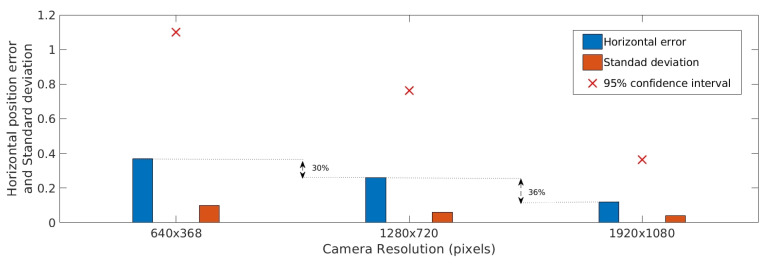
Absolute horizontal accuracy estimation for different camera resolution.

**Figure 6:**
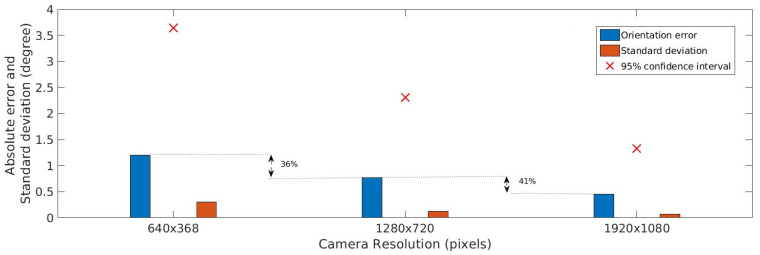
Horizontal orientation accuracy estimation for different camera resolutions.

**Figure 7:**
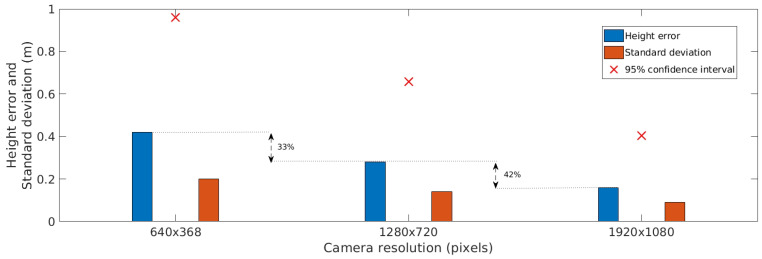
Height accuracy estimation for different camera resolutions.

As shown in Figure 5, camera resolution significantly influences accuracy. For example, an increase in resolution from 640×368 pixels to 1280×720 pixels decreases the position error by 30%. Orientation error is also significantly small for high-resolution images, as presented in Figure 6. In the case of height, as shown in Figure 7, it is possible to observe a reduction of, for example, 33% in the mean error for a 1280×720 pixel resolution compared with a 640×368 pixel resolution.

In addition, sunlight influenced the data position and orientation error as well. Direct and indirect sunny conditions did not significantly affect the measurements. However, cloudy conditions increased the positioning error by approximately 10%. A unique situation occurred in the presence of partial tag shadowing. Specifically, the detection of the tag was compromised because the algorithm could not effectively detect the border of the white and black external square of the tag. This situation must be avoided during an inspection operation.

### 4.2. Accuracy of UAV Flight Position Data Obtained Using the AR-Tag Reference

This subsection presents the results of the outdoor flight tests performed using the Bebop drone with the embedded RTK module and a fixed tag placed in the ground. The objective was to evaluate the tag position readings under the flight conditions and investigate the influences of the UAV displacement, camera vibration, and twist and detection distance limits on the readings.

The experiments were conducted in 10 flights performed at various heights. Post-processing calculations were performed to determine the accuracy of the collected information. The experiments were conducted on sunny days, with direct sunlight incident on the tag, between 10:00 and 17:00, at a maximum observed wind velocity of 10.0 km/h. The flight velocity was limited to 0.5 m/s. Tag size was 0.5 × 0.5 m. The images of the flight were collected at 640 × 368 pixels from the Bebop autonomy video transmission and at 1280 × 720 pixels from the Bebop drone on-board recording.

**Figure 8:**
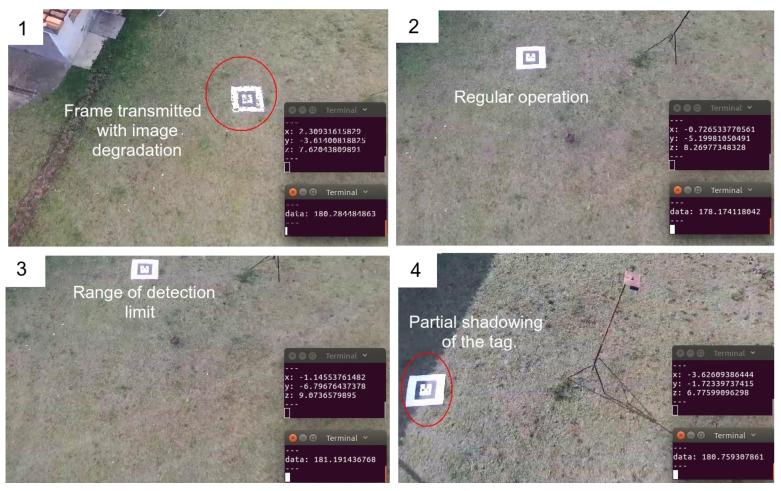
Figure showing snapshots of a flight round of the Bebop drone capturing the AR-Tag positioning. (**1**) Image frame transmitted with error, causing lost of accuracy; (**2**) Regular operation; (**3**) Tag near of detection limit; (**4**) Partial shadowing of the tag, causing lost of accuracy;

**Figure 9:**
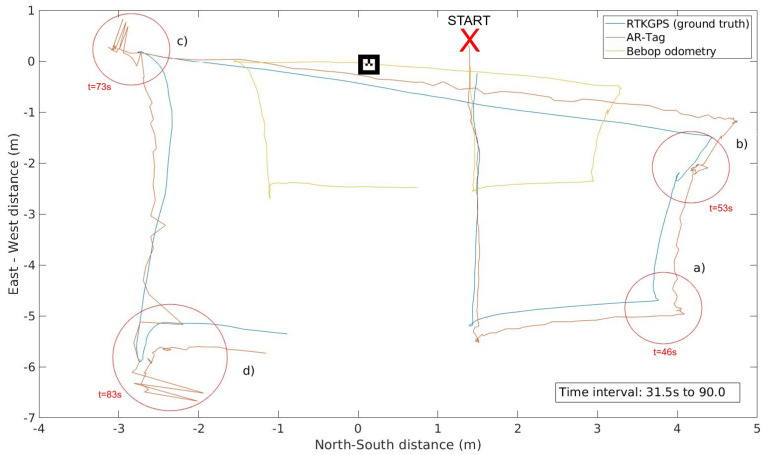
UAV flight path comparing the AR-Tag error with the Bebop odometry error.

Emild RTK-GPS data running in *FIX* mode, which presents a position accuracy of at least 1.0 cm, provided the ground truth for the position readings from Bebop odometry and AR-Tag. Figure 8 presents snapshots of a flight showing the tag image captures and the position readings. Figure 9 shows the flight path and the comparison of the collected data with the ground truth.

**Figure 10:**
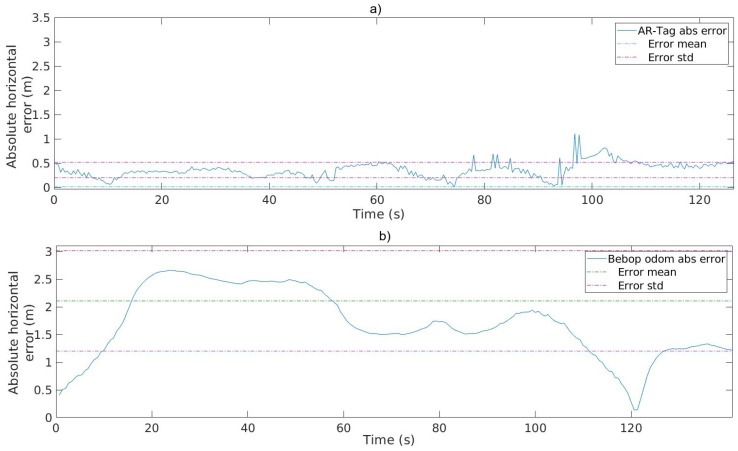
Absolute horizontal error for the AR-Tag and Bebop odometry with the mean and standard deviation values. (**a**) AR-Tag absolute error statistics; (**b**) Bebop odometry error statistics.

The blue, red, and yellow lines represent the ground truth, AR-Tag position, and Bebop drone odometry data, respectively. Compared with the odometry data, the AR-Tag presents significantly smaller error, thereby demonstrating the potential of this tool in providing proper position data for the UAV flight. Figure 10 presents the absolute horizontal error of the Bebop odometry and AR-Tag for the same flight.

The position error increases with the absolute distance from the tag, which is a vulnerability of the method. To ensure suitable implementation of the position control algorithms, the maximum distances between the UAV and tag must be maintained within a certain limit in addition to the safe levels. Compared with that of the regular GPS, the accuracy of the proposed method is significantly higher, demonstrating its promising potential for use in practical application UAV positioning systems. The results indicate that under normal conditions, the AR-Tag position’s accuracy corresponds to the inspection flight parameters defined for the project.

The flight direction change and stop points must be focused on because of the camera twist movement, as shown in the highlighted region a–c in Figure 9. Although the Bebop gimbal can correct most of the camera’s twists and vibrations during the flight, under these particular conditions, the drone’s horizontal twist until stabilization generates critical noise in the position readings, as highlighted by the red-circled points in the graph. Avoiding abrupt direction changes and halts can help minimize this effect. The highlighted region (d) also exhibits an increase in the position error. In the experiment, at this point, the tag was beyond the camera cover, indicating that it is essential to maintain the flight in a proper area to avoid loss of tag visibility.

Another critical point to be considered is the image degradation and loss of image frames because of the transmission to the base station, considering that this computer executes all the processing. A regular Wi-Fi connection produces this kind of loss, and the AR-Tag algorithm does not process the position in this situation correctly. Embedded processing in the UAV is recommended, leaving the base station only for the operational control. The Bebop drone does not present powerful embedded hardware and does not allow one to embed an external processing platform such as Raspberry-Pi because of payload limitations. A new UAV with a higher payload is being built to enable these experiments.

Table 4 presents the values of the mean and standard deviation for the absolute position errors obtained in all the flight tests. The calculations exclude the position readings from distances larger than the detection limit and those with camera twist noise. It is possible to verify that for all flight rounds the absolute error for the AR-Tag readings is considerably smaller than the Bebop odometry readings. Analyzing the confidence interval, Bebop odometry presents an accuracy range of 1.09±2.86 m against 0.45±0.86 m for AR-tag for low-resolution video and 0.25±0.75 m for high-resolution video. The aircraft’s IMU is highly sensitive to flight dynamic history, leading to cumulative errors. This is an advantage for the AR-Tag solution, which is not dependent on the flight history once there is a fix reference on the ground.

The position data must have higher accuracy than the limits of operation for the autonomous flight defined for the project, as described in Figure 3. Considering these values, the UAV must maintain the flight inside the inspection range from 3.0 to 5.0 m from the pylon. Analyzing the confidence interval shown in Table 4 for the low-resolution image capture, the position error remains inside a total range error of 2.07 m. For the high-resolution image capture, the range error was 1.75 m. These values assure us that the UAV flight will stay inside the inspection range, meaning that the obtained accuracy is proper. Compared with regular GPS accuracy, about 4.0 m, the proposed method presents a significant accuracy gain, showing good potential to build UAV positioning systems for practical applications.

### 4.3. UAV Horizontal Orientation Accuracy

The horizontal orientation is a key parameter to maintain the correct heading of the UAV during flight. Navigation hardware commonly includes a compass sensor with a standard accuracy of $0.5^{\circ }$; however, the presence of metal structures and magnetic fields from the power line pylon may lead to sensor interference. Using the AR-Tag orientation data can help enhance the accuracy of yaw angle orientation, mainly in static flight situations.

**Figure 11:**
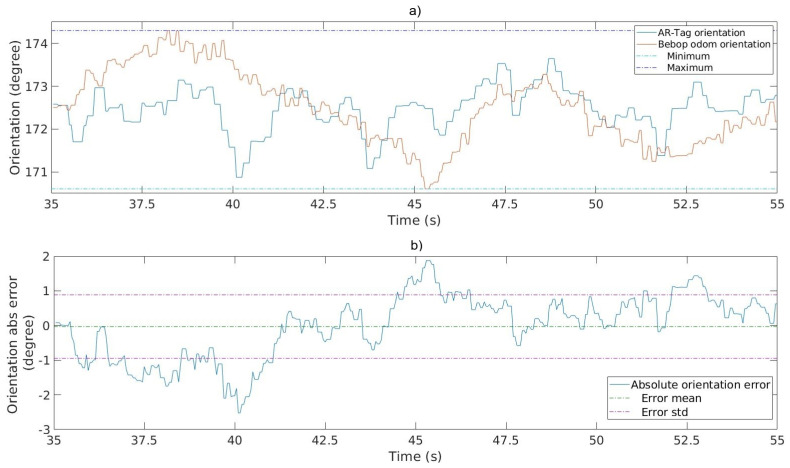
Horizontal orientation estimation. (**a**) AR-Tag vs. Bebop odometry orientation. (**b**) Absolute Bebop vs. AR-Tag orientation error.

An experiment was performed to evaluate the influences of the dynamic flight conditions on the horizontal orientation readings. The Bebop drone odometry orientation data provided the ground truth for the measurements. The tag size used in the experiment was 0.5 × 0.5 m. Ten flight tests were conducted at different heights, in a straight line above the tag. Images were collected in two different resolutions, 640×368 pixels and 1280×720 pixels. To minimize the dynamic flight influence on the Bebop odometry orientation data measurements, the values pertaining to the beginning and end of the displacements were discarded to ensure a proper stabilization time for the UAV IMU. Figure 11a shows the AR-Tag orientation versus Bebop odometry orientation readings for a 55.0 s flight interval. Figure 11b shows the absolute orientation error for the same interval, using the Bebop odometry orientation as the ground truth.

The graphs indicate that the AR-Tag orientation measurement readings were similar to those of the Bebop odometry. These results demonstrate the feasibility of using the tag to provide orientation data for the drone flight.

Table 5 presents the results of the absolute error in the measurements of 10 flights. The confidence interval values indicate that the absolute error remains within $0.93\pm 1.32^{\circ }$ for the low-resolution image processing and $0.55\pm 0.73^{\circ }$ for the high-resolution image processing, which is satisfactory in both cases. Even though the commercial compass sensor provides $0.5^{\circ }$ accuracy, the values obtained using AR-Tag are still acceptable, mainly because this data are used only in small displacements of the UAV and static flight heading. An accuracy of $3.57^{\circ }$ (95% confidence interval range) will create a horizontal position error of 0.62 m for a straight line displacement of 10.0 m executed by the UAV when a pylon segment is being inspected. It is essential to remember that this orientation angle is used to increase the robustness of the orientation data used in the flight, when the power line’s magnetic fields create significant compass sensor noise.

### 4.4. AR-Tag Height Accuracy Experiments

The accuracy of the UAV height must be ensured to realize autonomous flight near the pylon while preventing collision with cables and structural parts. The Bebop drone uses an ultra-sound sensor with a range of 8.0 m for the height readings, along with an IMU barometer sensor.

**Figure 12:**
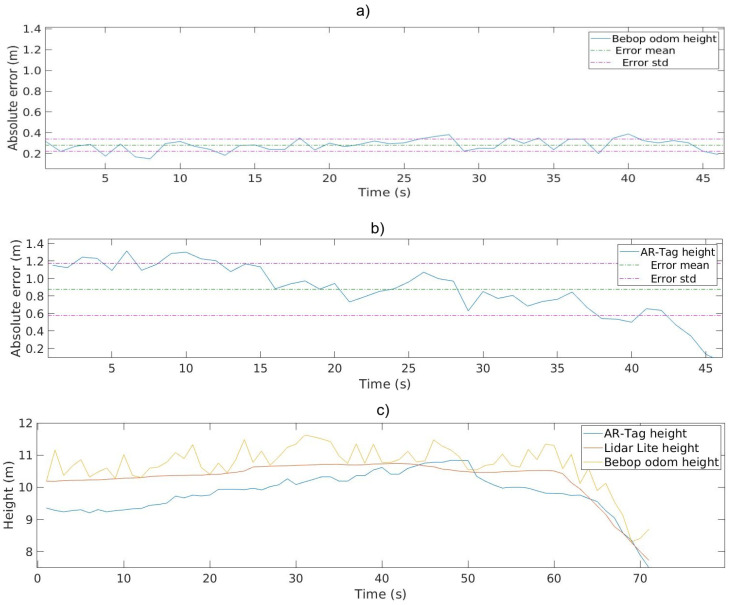
Height evaluation. (**a**) Bebop odometry height error; (**b**) AR-Tag height error; (**c**) height data from the AR-Tag, Bebop, and LIDAR-Lite compared.

To provide a high-accuracy ground truth for the height experiments, a LIDAR-Lite [37] sensor was attached to the Bebop base, pointing to the ground. LIDAR-Lite is a small laser pointer with a range of 40.0 m and accuracy of 10.0 cm with satisfactory performance in outdoor light conditions. The tag size used in the experiment was 0.5×0.5 m. Ten experiments were conducted with two different image resolutions, 640×368 pixels and 1290×720 pixels. The Bebop drone was manually commanded to take-off and move in a straight line to avoid aircraft frame twists that could influence the LIDAR sensor measurements. The AR-Tag and Bebop odometry height readings were recorded simultaneously using LIDAR-Lite. Figure 12a,b shows the error measurements of the AR-Tag and Bebop odometry height variables against the LIDAR-Lite. Figure 12c shows the height values obtained using the AR-Tag algorithm, Bebop odometry, and LIDAR-Lite ground truth, and their comparison for the same flight—a landing operation in this example.

Table 6 presents the means and standard deviations for the absolute height error for Bebop odometry data and the AR-Tag data calculated for all the flights. The range of operation for the height during an inspection was set to 2.0 m in the project parameter definition, as shown in Figure 3. Evaluation of the confidence interval for low-resolution image captures shows a height error range of 0.60±1.27 m, which is unsuitable for this study’s purposes. The AR-Tag error measurements show a considerable increase in error for high heights. This error level likely occurred because the AR-Tag algorithm could not effectively extract and calculate the tag size with reasonable accuracy at long distances. In addition to the tag position, the height depends on the square dimension being clearly defined, which is difficult to realize in this situation. The confidence interval analyses for the high-resolution collected data indicate that the height readings offer an accuracy better than 0.24±0.71 m, which is a proper result for the desired application.

### 4.5. UGV Position Accuracy Using UAV/RTK Position Data

This subsection describes the UGV displacement control experiment performed using the AR-Tag position reference provided by the UAV camera. The objective was to investigate the capability of the ground vehicle in precisely reaching a position setpoint, given the data provided by the UAV/AR-Tag schema.

**Figure 13:**
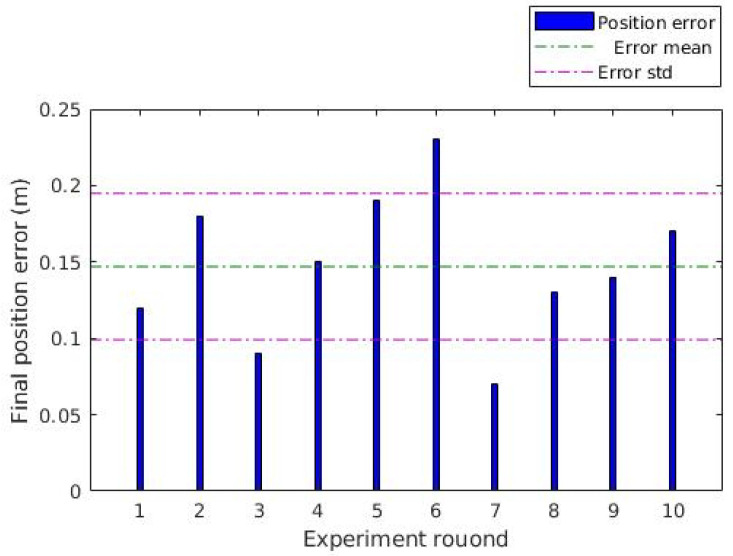
Position error of the unmanned ground vehicle (UGV) displacements: Position error of indoor experiment.

The first experiment evaluated the control of the UGV using only the AR-Tag position information in a controlled condition. The execution was done in an indoor site, with the Bebop drone fixed in support at 3.0 m height and a 20.0 cm tag placed at the top of the UGV. The vehicle began at a distance 2.0 m from the horizontal position of the UAV, in various directions. It was commanded to move and reach the final point, stopping at a 0.1 m tolerance interval. For each round, the final position error was manually measured. The total error of each round is presented in Figure 13. The vehicles reached the final point in all rounds successfully. As shown in the graph, the position error presented by the cooperative positioning was less than 25.0 cm. Considering the tolerance of 10.0 cm, the results show that the control system shows good performance in controlled conditions.

**Figure 14:**
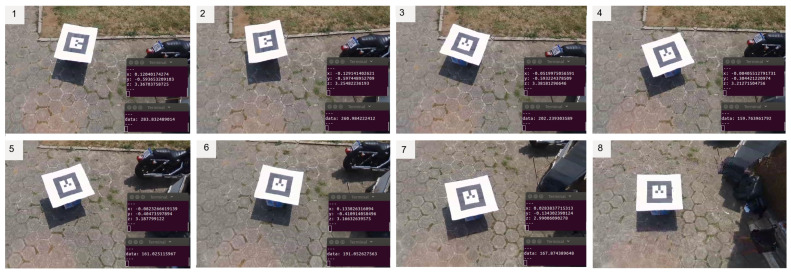
Snapshots of the UGV control displacement using AR-Tag position data in an outdoor site. (**1**) Start of the UGV displacement; (**2**)–(**7**) The UGV is following the UAV; (**8**) The UGV reach the position below the UAV;

The next experiment investigated the influences of outdoor flight conditions on the autonomous control of the UGV. Some factors such as camera vibration, drone twists, missing of image frames, and variation of light can affect the control algorithm. Ten experiment rounds were performed with different start points. The UAV was commanded to take-off and stay in a static position above the UGV displacement area. In each round, the UGV reached the setpoint and stopped at the tolerance distance. At the end of each displacement, the total position error was measured to examine the accuracy using an image of the final point reached by the robot. Figure 14 shows snapshots of a displacement round.

**Figure 15:**
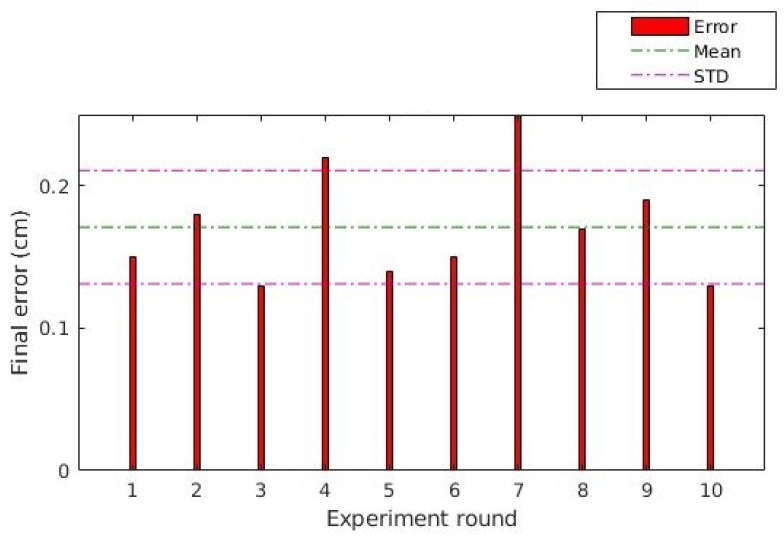
Position error of the UGV displacements: position error of the outdoor experiment.

Figure 15 presents the absolute position error for all the rounds. The error was calculated by measuring the absolute distance of the central point of the UGV top compared with the geometric center of the image. For all the displacements, the final error was less than 0.25 m with 0.2 m standard deviation for the group. This error dimension is small compared with the size of the vehicle and the total distance of the executed path, allowing us to conclude that the control position is acceptable for the proposed method.

## 5. Conclusions

This paper reports on the general concept of a cooperative inspection architecture with a UGV and a UAV to create a positioning system for power pylon inspection applications. In this work, the objective was to evaluate a redundant positioning proposal based on the cooperative position reference data obtained using AR-Tags. Real-world experiments were conducted to confirm the feasibility of the arrangement.

UAV flight position experiments showed adequate results in horizontal position data readings for regular flight operation, with respect to the distance limits. The orientation data confirmed proper performance for the required application. While the height error was inadequate because of the inaccuracy in the 640×368 pixel resolution images captured in the flight experiments, the accuracy obtained using 1280×720 pixel resolution video was appropriate. Fusing the height data with other sensor data, such as LIDAR and IMU, could improve the height data accuracy to optimal levels.

The UGV positioning experiments were successful, both in controlled and in outdoor environments, indicating that the AR-Tag solution is viable for providing a simple and effective solution for such applications. The positioning error generated by the algorithm is small, and the robustness of the control using the AR-Tag data is adequate.

A limitation of this approach corresponds to the large tag required to inspect energy pylons at a large height; for instance, the tag size must be larger than 1.0 m for a pylon height of 25.0 m pylon height. Installing such a large tag in the ground vehicle may be challenging. The AR-Tag tool in this study was selected to allow rapid algorithm implementation using the available package. Visual tags with a higher performance, such as active light tags, may be used to substitute the AR-Tag.

This paper presents the first part of a more extensive power distribution inspection system. In general, the inspection process of power pylons is challenging owing to the highly accurate position control required to ensure safe operation. In addition, certain other aspects must be investigated to practically implement the collaborative inspection system in future works, such as the autonomous landing of the UAV at the UGV base, power line segment following, ground path following, UAV and UGV intelligent obstacle avoidance, long-range communication among the agents, inspection data treatment, and data fusion. Our group is actively working to develop solutions for these aspects and other technical challenges.

## Figures and Tables

**Table 1:** Related works.

Paper	Detailed Pylon Inspection	Vision-Based Positioning	AR/Tag Positioning	Cooperative Inspection
[10,11]	Yes	Yes	No	No
[13,14,15,29]	No	Yes	Yes	No
[16]	Yes	Yes	Yes	No
[21,23,27,28]	No	Yes	Yes	No
Proposed approach	Yes	Yes	Yes	Yes

**Table 2:** Inspection parameters defined for the project.

Parameter	Value
Recommended distance range for image data collection	4.0 ± 1.0 m
Critical safe flight distance from structure obstacles	2 m
Average UAV flight velocity during pylon inspection	0.5 m/s
Average UAV flight velocity during power line segment following	1.5 m/s
Range of UAV inspection around the tower	360${}^{\circ }$
Typical inspected power pylon height	20–50 m
Horizontal displacement between the UAV and the UGV on a flight	5.0 m

**Table 3:** Means and standard deviations for absolute horizontal error, height error, and orientation error read in the static experiment.

	Distance (m)		2.0		6.0		10.0		14.0		18.0		22.0	
Camera. (Pixels)	Tag Size (cm)		Mean Error	Std	Mean Error	Std	Mean Error	Std	Mean Error	Std	Mean Error	Std	Mean Error	Std
640 × 368	50.0	Hor. Err	0.12	0.02	0.23	0.06	0.37	0.10	o.r	-	o.r	-	o.r	-
		Height Error	0.10	0.01	0.25	0.05	0.37	0.09	o.r	-	o.r	-	o.r	-
		Orient. Error	0.51	0.02	0.83	0.09	1.20	0.12	o.r	-	o.r	-	o.r	-
	90.0	Hor. Err	0.05	0.01	0.12	0.02	0.23	0.06	0.35	0.10	0.46	0.12	o.r	-
		Height Error	0.08	0.01	0.13	0.03	0.22	0.07	0.28	0.10	0.35	0.12	o.r	-
		Orient. Error	0.32	0.01	0.51	0.07	0.93	0.09	1.31	1.11	1.52	1.20	o.r	-
1280 × 720	50.0	Hor. Err	0.08	0.01	0.18	0.02	0.26	0.04	0.43	0.33	o.r	-	o.r	-
		Height Error	0.09	0.01	0.17	0.01	0.26	0.07	0.55	0.45	o.r	-	o.r	-
		Orient. Error	0.34	0.01	0.51	0.07	0.77	0.19	1.24	0.38	o.r	-	o.r	-
	90.0	Hor. Err	0.03	0.01	0.10	0.01	0.18	0.02	0.22	0.05	0.38	0.09	o.r	-
		Height Error	0.06	0.01	0.10	0.01	0.16	0.03	0.23	0.04	0.31	0.10	o.r	-
		Orient. Error	0.30	0.01	0.37	0.03	0.44	0.08	0.75	0.87	1.66	1.00	o.r	-
1920 × 1080	50.0	Hor. Err	0.04	0.01	0.06	0.01	0.09	0.02	0.12	0.05	o.r	-	o.r	-
		Height Error	0.05	0.02	0.08	0.01	0.12	0.03	0.17	0.05	o.r	-	o.r	-
		Orient. Error	0.21	0.02	0.39	0.03	0.45	0.08	0.97	0.10	o.r	-	o.r	-
	90.0	Hor. Err	0.01	0.01	0.03	0.01	0.09	0.02	0.18	0.05	0.28	0.07	0.36	0.12
		Height Error	0.01	0.01	0.05	0.01	0.12	0.04	0.20	0.07	0.27	0.09	0.39	0.12
		Orient. Error	0.05	0.01	0.22	0.02	0.49	0.08	0.64	0.12	1.33	0.90	1.45	1.00

Horr.Err: absolute horizontal position error (m); Height Error: absolute height error (m); Orient.Error: absolute horizontal error (degree); [Std]: standard deviation; [o.r]: out-of-range

**Table 4:** Horizontal absolute error measurement statistics: AR-Tag × Bebop odometry.

Flight	(1280 × 720) AR-Tag Position Error Mean (m)	AR-Tag Position Standard Deviation	(640 × 368) AR-Tag Position Error Mean (m)	AR-Tag Position Standard Deviation	Bebop Position Error Mean (m)	Bebop Position Error Standard Deviation
1	0.24	0.21	0.50	0.30	0.80	0.90
2	0.25	0.23	0.44	0.22	0.75	0.81
3	0.19	0.33	0.47	0.27	2.42	1.10
4	0.29	0.35	0.41	0.33	0.57	0.99
5	0.22	0.32	0.52	0.31	0.43	1.09
6	0.27	0.28	0.60	0.32	1.20	1.15
7	0.20	0.37	0.45	0.36	0.93	1.22
8	0.31	0.21	0.33	0.35	2.38	1.33
9	0.32	0.25	0.31	0.29	1.47	1.10
10	0.24	0.29	0.47	0.23	2.99	0.93
**Mean values**	0.25	0.28	0.45	0.30	1.19	1.06
**Mean error superior 95% confidence limit (M)**	0.25 ± 0.75		0.45 ± 0.81		1.09 ± 2.86	

**Table 5:** Absolute orientation statistics for 10 flights.

Flight	(1280 × 720) Absolute Orientation Error	Standard Deviation	(640 × 368) Absolute Orientation Error	Standard Deviation
1	0.67	0.23	1.10	0.52
2	0.55	0.30	1.04	0.58
3	0.73	0.31	0.79	0.43
4	0.56	0.25	0.86	0.45
5	0.59	0.27	0.88	0.52
6	0.47	0.20	0.93	0.49
7	0.51	0.33	0.78	0.58
8	0.57	0.25	1.02	0.37
9	0.43	0.24	0.99	0.45
10	0.39	0.28	0.93	0.56
Mean	0.55	0.27	0.93	0.49
**95% Confidence interval (degree)**	0.55 ± 0.73		0.93 ± 1.32	

**Table 6:** Absolute error height measurement: AR-Tag × Bebop odometry.

Height	(1280 × 720) AR-Tag Height Error Mean (m)	AR-Tag Error Standard Deviation	(640 × 368) AR-Tag Height Error Mean (m)	AR-Tag Error Standard Deviation	Bebop Height Error Mean (m)	Bebop Heigh Error Standard Deviation
3.0	0.12	0.19	0.30	0.32	0.12	0.07
4.0	0.15	0.22	0.38	0.27	0.15	0.09
5.0	0.16	0.24	0.42	0.35	0.22	0.10
6.0	0.19	0.25	0.51	0.44	0.19	0.14
7.0	0.22	0.23	0.52	0.47	0.25	0.13
8.0	0.26	0.26	0.65	0.51	0.37	0.15
9.0	0.29	0.30	0.74	0.56	0.43	0.18
10.0	0.33	0.31	0.80	0.55	0.48	0.19
11.0	0.36	0.27	0.83	0.59	0.57	0.22
12.0	0.37	0.39	0.87	0.63	0.59	0.23
**Mean values**	0.24	0.27	0.60	0.47	0.33	0.15
**Mean error superior 95% confidence limit (m)**	0.24 ± 0.71		0.60 ± 1.27		0.33 ± 0.40	

## Supplementary Materials

A video presenting the experiments is available online at https://youtu.be/pPI6zcfIYzI.

## Abbreviations

The following abbreviations are used in this manuscript:

UGV	Unmanned Ground Vehicle
UAV	Unmanned Aerial Vehicle
LIDAR	Light Detection Additionally, Ranging
RTK-GPS	Real-Time Kinematic Global Positioning System
